# Portanini (Insecta: Hemiptera: Cicadellidae) from Peru: checklist with new records and descriptions of two new species

**DOI:** 10.7717/peerj.10222

**Published:** 2020-12-11

**Authors:** Jádila Santos Prando, Clayton Corrêa Gonçalves, Daniela Maeda Takiya

**Affiliations:** 1 Laboratório de Entomologia, Departamento de Zoologia, Instituto de Biologia, Universidade Federal do Rio de Janeiro, Rio de Janeiro, RJ, Brazil; 2 Programa de Pós-graduação em Biodiversidade e Biologia Evolutiva, Instituto de Biologia, Universidade Federal do Rio de Janeiro, Rio de Janeiro, RJ, Brazil

**Keywords:** Checklist, Leafhoppers, Biodiversity, Neotropics

## Abstract

Portanini Linnavuori, 1959 is a small tribe of neotropical leafhoppers that includes two genera: *Portanus* Ball, 1932 and *Metacephalus* DeLong & Martinson, 1973. Herein, a checklist of portanines from Peru is given, including several new species records for the country, elevating the known diversity from nine to 22 species. In addition, four species have their department ranges expanded in Peru. Two new portanine species are also described: *Metacephalus mamaquilla* **sp. nov.** and *Portanus tambopata* **sp. nov.** both from Tambopata National Reserve, Madre de Dios, Peru and we make available habitus photos of other Portanini species from this reserve.

## Introduction

The hemipteran infraorder Cicadomorpha comprises approximately 35,000 described species of plant sap-sucking insects distributed worldwide (Dietrich, 2005). It includes the superfamily Membracoidea that comprises the treehoppers (Membracidae, Aetalionidae, and Melizoderidae) and leafhoppers (Cicadellidae and Myerslopiidae) (Deitz & Dietrich, 1993). With approximately 21,000 species, 2,550 genera and 25 subfamilies, Cicadellidae is the largest hemipteran family, being cosmopolitan in distribution, occurring everywhere plants (their hosts) can survive (Dietrich, 2013; Bartlett et al., 2018).

Included in the subfamily Aphrodinae by Dietrich (2005), Portanini was erected by Linnavuori (1959) as one of the leafhopper tribes restricted to the Neotropical region. Portanines can be recognized by their long and slender bodies; their crown triangularly produced; their ocelli on anterior margin of head, distant from the eyes; and the antennae unusually long (Linnavuori, 1959; Felix & Mejdalani, 2016). Currently, the tribe include 63 valid species divided into two genera: *Portanus* Ball, 1932 and *Metacephalus* DeLong & Martinson, 1973 with 49 and 14 valid species, respectively (Felix & Mejdalani, 2016; Souza, Takiya & Felix, 2017; Carvalho & Cavichioli, 2017; Freytag, 2017; Felix et al., 2020). Members of *Metacephalus* can be distinguished from *Portanus* by the following set of male features (Carvalho & Cavichioli, 2009): (1) pygofer strongly produced posteriorly, usually with a pair of spiniform processes on posteroventral margin (pygofer slightly produced and with variable posterior margin in *Portanus*); (2) subgenital plates triangular, without transverse unpigmented line at basal third (subgenital plates with transverse unpigmented line at basal third in *Portanus*); and (3) connective V-shaped (T-shaped in *Portanus*).

The leafhopper fauna of the Neotropical region is still poorly known. Approximately 5,000 species are described, but there can easily be 5,000–10,000 undescribed species in the region, and perhaps many more (Freytag & Sharkey, 2002). Peru has one of the most megadiverse leafhopper faunas in the Neotropical region with currently 634 species of which only nine species of Portanini are recorded (Linnavuori, 1959; DeLong & Martinson, 1973; DeLong & Linnavuori, 1978; DeLong, 1980, 1982; Lozada, 1992; Carvalho & Cavichioli, 2009; Costa & Lozada, 2010; Felix & Mejdalani, 2016; Souza, Takiya & Felix, 2017).

In this article, a checklist of Portanini from Peru is provided, including eleven new country records, elevating the diversity of known Peruvian portanines from nine to 22 species and four species have their distribution expanded in the country. Additionally, two new species of Portanini from Tambopata National Reserve (Madre de Dios, Peru) are described and illustrated and habitus photos of the 10 Portanini species identified from this reserve are also provided.

## Materials and Methods

Specimens studied are deposited in the following collections: Museo de Historia Natural, Universidad Nacional Mayor de San Marcos, Lima (MUSM); Coleção Entomológica Prof. José Alfredo Pinheiro Dutra, Instituto de Biologia, Universidade Federal do Rio de Janeiro, Rio de Janeiro (DZRJ); and Insect Collection, Illinois Natural History Survey, Champaign (INHS). Labels of type material are quoted separately, line breaks are indicated by a backslash (\) and additional information given between brackets ([ ]).

For species identification, male genitalia were prepared following Oman (1949), where the abdomen is cleared in 10% KOH hot solution for some minutes and washed for a short time in water. For the female genitalia, the protocol from Zanol (1988) was used, in which the abdomen is cleared in 10% KOH at room temperature for nearly 15 h and washed with distilled water for 15 min. Observation and dissection of genital parts were conducted in glycerin. Structures were observed and photographed with a Leica M205C stereomicroscope equipped with a Leica DFC450 digital camera attached. Photographs at different focal planes were stacked with the software Leica Application Suite and edited in Adobe Photoshop^®^. Studied genital structures were preserved in glycerin within microvials attached to the specimens. Morphological terminology mostly follows Dietrich (2005), while female valvulae terminology follows Hill (1970).

The electronic version of this article in Portable Document Format (PDF) will represent a published work according to the International Commission on Zoological Nomenclature (ICZN), and hence the new names contained in the electronic version are effectively published under that Code from the electronic edition alone. This published work and the nomenclatural acts it contains have been registered in ZooBank, the online registration system for the ICZN. The ZooBank LSIDs (Life Science Identifiers) can be resolved and the associated information viewed through any standard web browser by appending the LSID to the prefix http://zoobank.org/. The LSID for this publication is: [urn:lsid:zoobank.org:pub:EEA39E0C-D2C0-494C-B1D7-F7E6B3D818CD]. The online version of this work is archived and available from the following digital repositories: PeerJ, PubMed Central and “CLOCKSS.”

## Results

**Species descriptions**

***Metacephalus*DeLong & Martinson, 1973**

*Metacephalus* DeLong & Martinson, 1973: 225. Type species: *M. albocrux* DeLong & Martinson, 1973.

*Paraportanus* Carvalho & Cavichioli, 2009: 26. Type species: *P. jenniferae* Carvalho & Cavichioli, 2009 [synonymized by Souza, Takiya & Felix, 2017].

*Metacephalus mamaquilla* **sp. nov.**

urn:lsid:zoobank.org:act:8CD03270-1760-4962-8D9E-26F639FB8E04

(Figs. 1–2)

**Figure 1:**
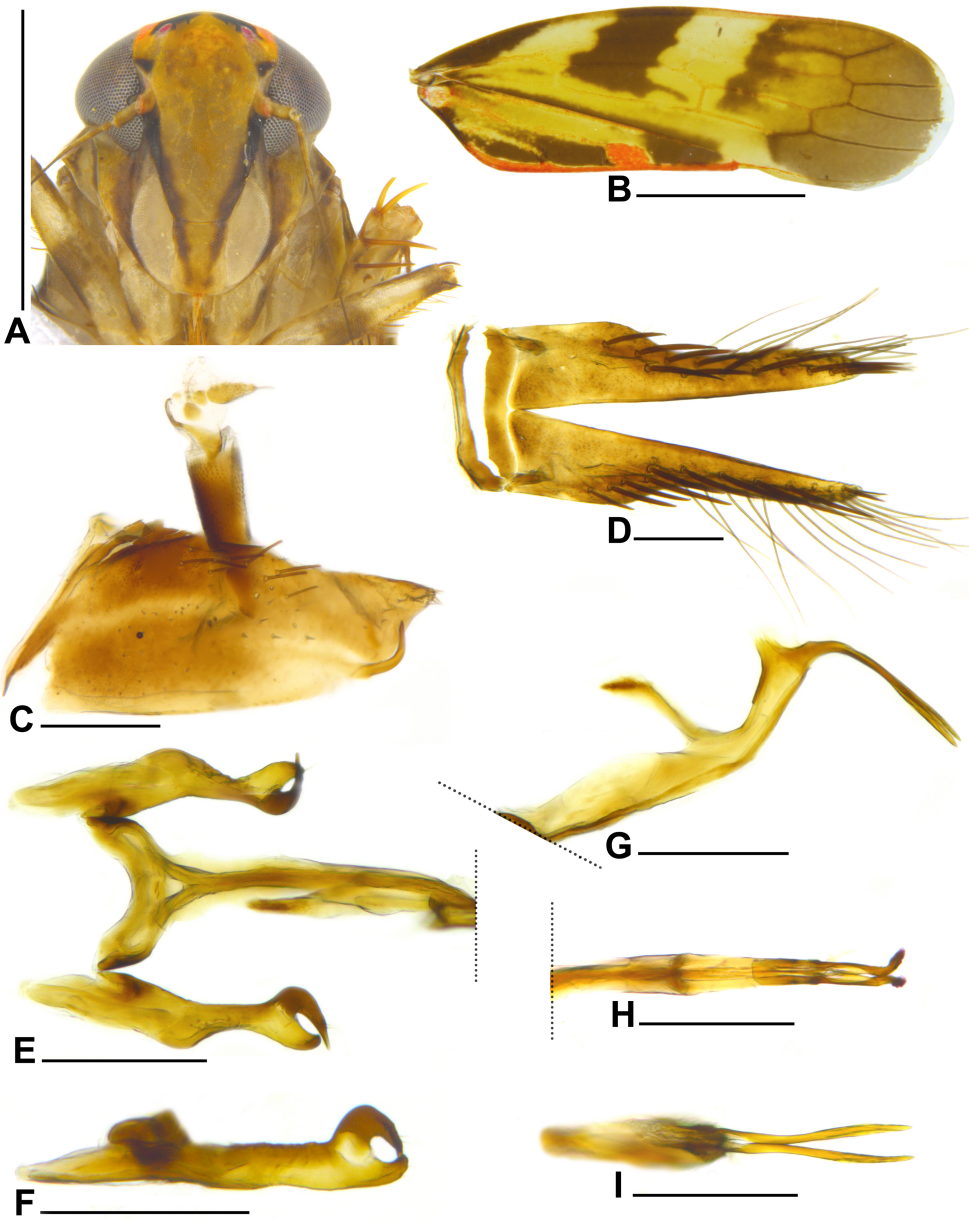
*Metacephalus mamaquilla* sp. nov., male holotype. (A) Head and anterior thorax, ventral view. (B) Forewing, dorsal view. (C) Pygofer and anal tube, lateral view. (D) Valve and subgenital plates, ventral view. (E) Connective and styles, dorsal view. (F) Style, lateral view. (G) Aedeagus, lateral view. (H) Aedeagus, dorsal view. (I) Aedeagus, posterior view. Scale bars: (A and B) 1 mm; (C–I) 0.2 mm. Photo credit: Clayton C. Gonçalves.

**Figure 2:**
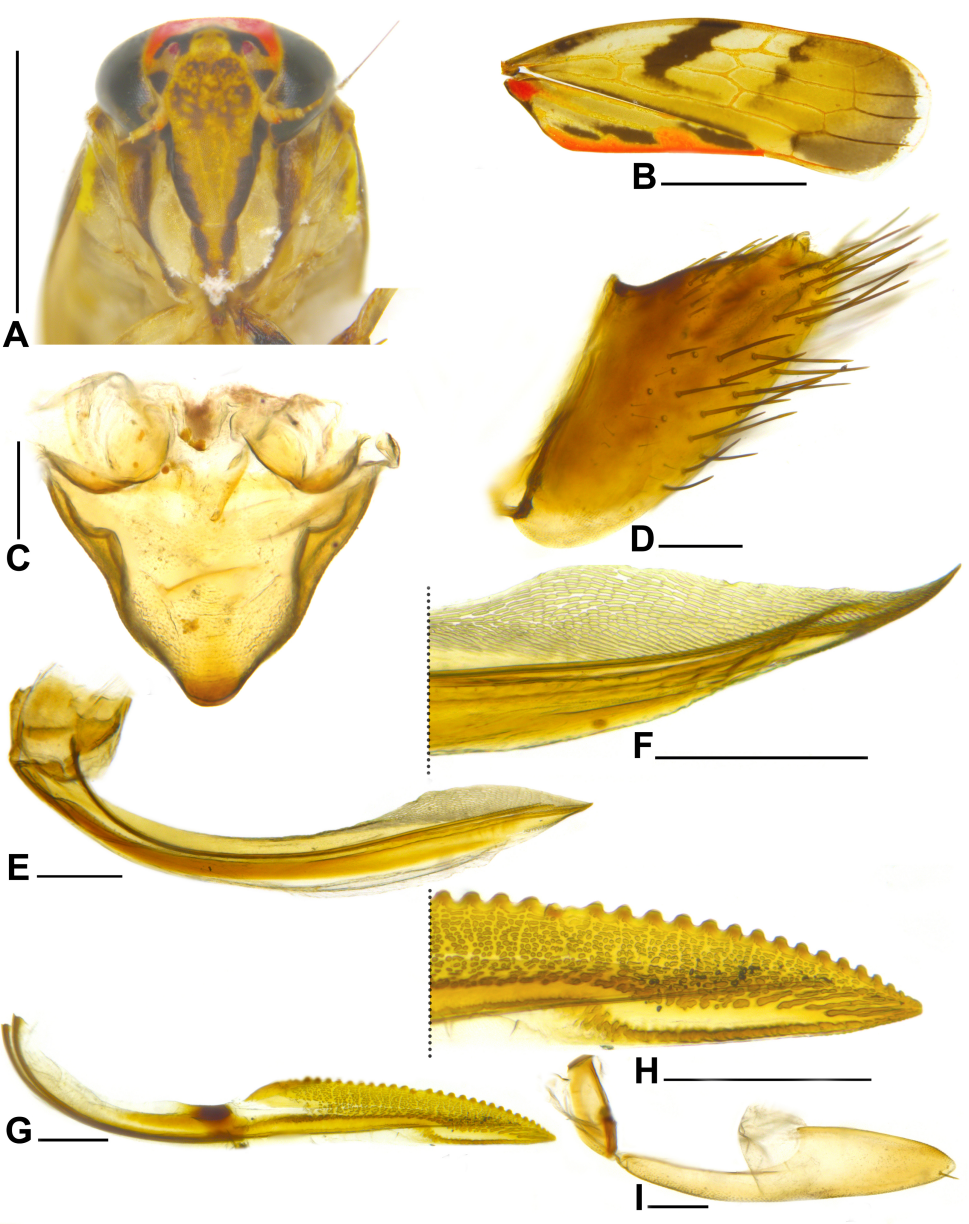
*Metacephalus mamaquilla* sp. nov., female paratype. (A) Head and anterior thorax, ventral view. (B) Forewing, dorsal view. (C) Sternite VII, ventral view. (D) Pygofer and anal tube, lateral view. (E) First valvifer and first valvula, lateral view. (F) Apical portion of first valvula, lateral view. (G) Second valvula, lateral view. (H) Apical portion of second valvula, lateral view. (I) Second valvifer and gonoplac, lateral view. Scale bars: (A and B) 1 mm; (C–I) 0.2 mm. Photo credit: Clayton C. Gonçalves.

**Type locality.** Refugio Amazonas, Madre de Dios, Peru.

**Diagnosis**. Male pygofer (Fig. 1C), in lateral view, subrectangular; posterior margin acute; with slender and acute preapical ventral process turned dorsally. Aedeagus (Figs. 1G–1I) apex with pair of long and slender divergent processes curved posteroventrally and with apices acute. Female sternite VII (Fig. 2C) subtriangular; lateral margins slightly sinuous and strongly convergent apically; posterior margin slightly convex.

**Measurements** (mm). Males (*n* = 13)/females (*n* = 5): body length, 5.5–6.0/5.9–6.3; crown length, 0.3–0.4/0.4–0.5; transocular width, 1.2–1.3/1.4; interocular width, 0.5–0.6/0.6; maximum pronotum width, 1.3–1.4/1.4–1.6; forewing length, 4.3–4.9/4.8–5.2.

**Coloration**. Crown mostly orange; apex with pale-yellow macula; anterior third with pair of black Y-shaped macula, each surrounding respective ocellus; posterior two-thirds with pair of short longitudinal parallel pale-yellow stripes; posterior margin with pair of black spots adjacent to eyes. Ocellus red. Face (Figs. 1A and 2A) ivory to pale yellow; lateral margin of frontoclypeus and anteclypeus dark brown; lorum (Figs. 1A and 2A) ivory; gena (Figs. 1A and 2A) mostly light-brown with outer margin pale yellow. Pronotum dark brown, with several ivory spots. Mesonotum orange; anterior margin and pair of lateral triangular maculae dark brown; short pale-yellow stripe on anterior half. Scutellum orange. Forewing (Figs. 1B and 2B) translucent brown; clavus with slender line along anal margin, large spot connected to line at apex of first anal vein and another at base, orange, additionally, three large dark-brown elongate maculae adjacent to orange longitudinal line; corium with slender brown line adjacent to claval suture, with three dark-brown maculae near costal margin: first small, near base, second forming broad oblique band extending close to Cu vein, and third forming oblique narrower band extending to base of inner anteapical cell. Thoracic venter ivory. Profemur with two large brown maculae, one larger at middle third and one smaller at apex; protibia pale yellow on dorsal surface and dark brown on ventral surface, setae dark brown; mesofemur with large brown subapical macula, mesotibia similar to protibia; metafemur pale yellow with slender brown stripe on dorsal surface, apex orange; metatibia pale yellow with brown areas, base orange; all tarsomeres pale yellow. Female: color pattern similar to male except for forewing with narrower darkbrown maculae (Fig. 2B).

**Description**. Head (Figs. 1A and 2A), in dorsal view, with anterior margin rounded; crown median length approximately half to eight-tenths of interocular width and three to four-tenths of transocular width; lateral frontal suture reaching ocellus; epicranial suture not extended to imaginary transverse line between ocelli; texture shagreen. Pronotum slightly wider than head; lateral margin angulate; dorsolateral carina conspicuous and complete; posterior margin straight; texture smooth. Mesonotum shagreen. Forewing (Figs. 1B and 2B) with distinct venation; three closed anteapical cells. Metatibia with rows AD and PD both with 10–11 long cucullate setae intercalated by 0–3 shorter cucullate setae; tibia apex with three platellae between pair of outer slightly longer cucullate setae; first tarsomere slightly longer than combined length of second and third; tarsomeres posterior margin with three, two, and zero platellae, respectively, between pair of outer slightly longer setae.

**Male genitalia**. Pygofer (Fig. 1C), in lateral view, longer than high; subrectangular; posterior margin acute; with few macrosetae distributed near dorsal margin and at apex; posteroventral margin with slender and acute ventral process turned dorsally. Valve (Fig. 1D), in ventral view, about three times wider than long; posterior margin sinuous. Subgenital plate (Fig. 1D) extending slightly beyond apex of pygofer; slightly upturned; in ventral view, surface with 11–14 robust macrosetae mostly uniseriate (some specimens have one or two additional macrosetae not aligned) and fine long microsetae. Connective (Fig. 1E), in dorsal view, Y-shaped; apex fuzed with aedeagus preatrium. Style (Figs. 1E and 1F) with apodeme (basal portion anterad of connective articulation) one-fifth of total length; apical fifth enlarged and appearing bifid due to elongate and robust preapical lobe; preapical lobe with few fine microsetae; preapical region sculptured; apex acute and curved outwards, bearing robust spine. Aedeagus (Figs. 1G–1I) with long preatrium; dorsal apodeme well developed, long and narrow; shaft tubular; apex with pair of long and slender divergent processes curved posteroventrally with apices acute. Anal tube segment X (Fig. 1C) with base conical and remainder tubular; with dentiform microsculpturing throughout.

**Female genitalia**. Sternite VII (Fig. 2C), in ventral view, as wide as long; subtriangular; lateral margins slightly sinuous and strongly converging apically; posterior margin convex. Pygofer (Fig. 2D), in lateral view, higher than long; subtriangular; ventral margin twice longer than dorsal margin; dorsal margin with concavity at apical third; macrosetae distributed on posterior two-thirds; some interspersed microsetae; apex angulate. First valvifer (Fig. 2E) subquadrangular. First valvula (Fig. 2E), in lateral view, expanded apically; ventral interlocking device located on basal fourth of blade; dorsal sculptured area on apical third, with sculpturing elongate derived from a strigate pattern (Fig. 2F); apex falciform. Second valvifer (Fig. 2I) about three times higher than long. Second valvula (Figs. 2G and 2H) with apical half expanded, narrowing to apex; dorsal margin with 28 separate teeth without denticles (single specimen dissected); duct area with maculose sculpturing; ventral margin without preapical prominence or denticles; apex acute. Third valvula (Fig. 2I), in lateral view, with basal half distinctly narrower than apical half; microsetae distributed along ventral margin and near apex on dorsal margin; two apical macroseta; apex narrowly rounded. Anal tube segment X (Fig. 2D), in lateral view, short, length one-third of dorsal margin of pygofer; basal half conical; apical half cylindrical.

**Remarks**. *Metacephalus mamaquilla* **sp. nov.** is similar to *Metacephalus facetus* (Kramer, 1961) and *Metacephalus sakakibarai* (Souza, Takiya & Felix, 2017) in the aspect of the paired apical aedeagus processes, which are long and divergent in caudal view. However, the new species can be distinguished from all other *Metacephalus* species by the following characteristics: (1) male pygofer (Fig. 1C) with posterior margin acute and preapical acute ventral process turned dorsally; and (2) aedeagus (Fig. 1G–1I) with shaft apex curved dorsally with pair of long, narrow and divergent processes curved posteroventrally.

**Etymology**. The species epithet is a homage to the Inca goddess Mama Quilla, considered a defender of women. The species epithet is treated as a noun in apposition.

**Material studied. Holotype**. 1 male, “**PERU**, MD [Madre de Dios], Albergue\Refugio Amazonas\12°52′30″[S]/69°24′35″[W]\231 m 20.ii.2016\J. Grados”, “WIRED AMAZON\PROJECT\PAN TRAP” (Cicadell-JGA-005, MUSM). **Paratypes**. 1 male, same data as holotype (DZRJ-AUCH-125); 1 male, same data as holotype, except “19.ii.2016” (Cicadell-JGA-003, MUSM); 1 male, same data as holotype, except “29.ii.2016” (Cicadell-JGA-006, MUSM); 1 male, same data as holotype, except “241 m 05.iii.2016\D. Couceiro” (MUSM); 1 male, same data as holotype, except “05.x.2016\D. Couceiro” (MUSM); 1 male, 2 females, same data as holotype, except “17.x.2016\D. Couceiro” (DZRJ-AUCH-122-124); 3 males, same data as holotype, except “06.xi.2016\D. Couceiro” (DZRJ-AUCH-126-128); 1 female, same data as holotype, except “241 m 02.iii.2017” (Cicadell-JGA-004, MUSM); 1 male, same data as holotype, except “241 m 04.iii.2017” (Cicadell-JGA-007, MUSM); 1 male, same data as holotype, except “241 m 10.iv.2017\D. Couceiro” (MUSM); 1 female, same data as holotype, except “241 m 20.iv.2017\D. Couceiro” (MUSM); 1 male, 1 female, same data as holotype, except “241 m 26.iv.2017\D. Couceiro” (MUSM).

***Portanus* Ball, 1932**

*Portanus* Ball, 1932: 18. Type species: *Scaphoideus stigmosus* Uhler, 1895.

*Portanus tambopata* **sp. nov.**

urn:lsid:zoobank.org:act: 9C799CBA-FD0C-4DB3-931D-7FB7ECA440E6

(Figs. 3–4)

**Figure 3:**
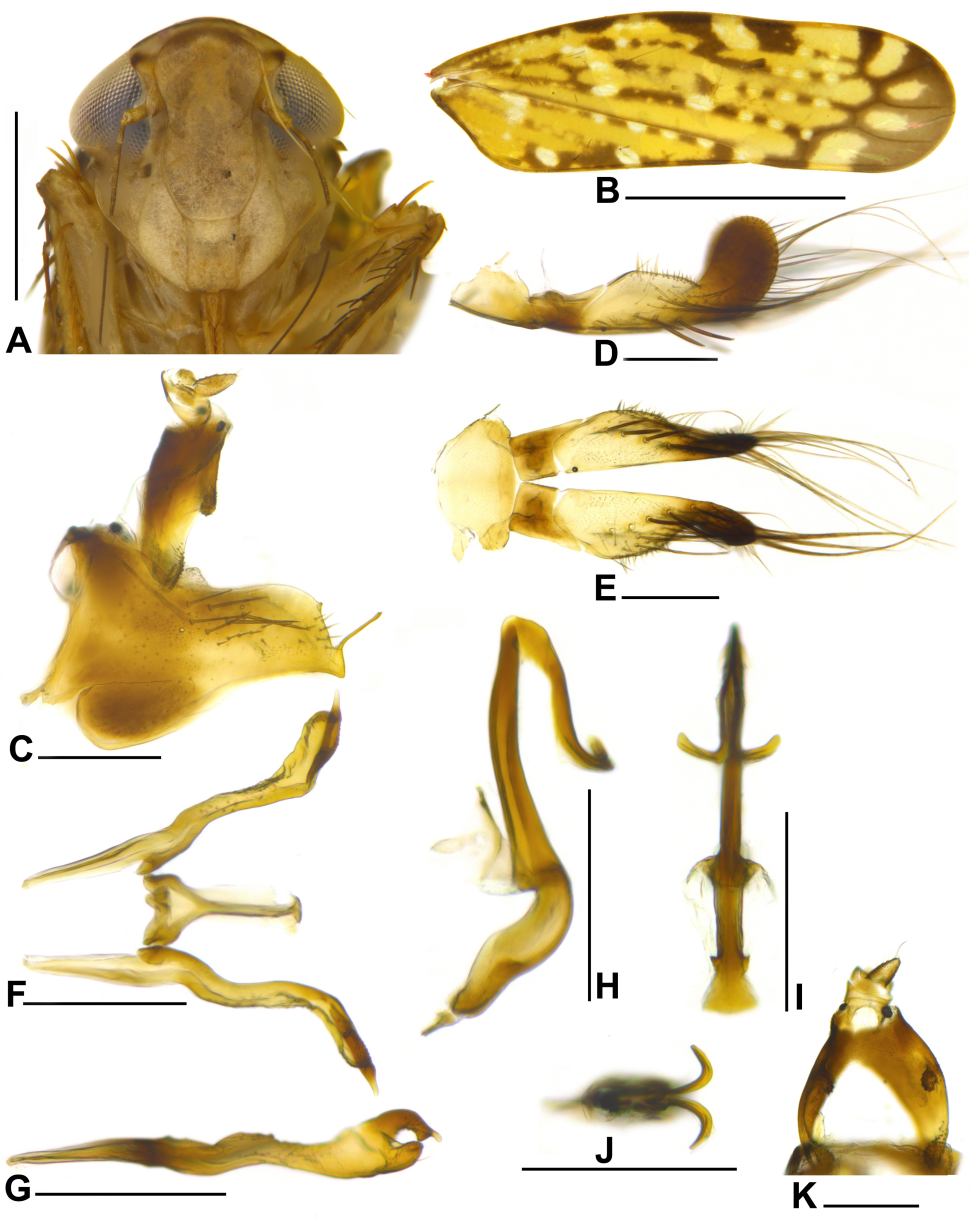
*Portanus tambopata* sp. nov., male holotype. (A) Head and anterior thorax, ventral view. (B) Forewing, dorsal view. (C) Pygofer and anal tube, lateral view. (D) Valve and subgenital plate, lateral view. (E) Valve and subgenital plates, ventral view. (F) Connective and styles, dorsal view. (G) Style, lateral view. (H) Aedeagus, lateral view. (I) Aedeagus, posterior view. (J) Aedeagus, dorsal view. (K) Anal tube, ventro-posterior view. Scale bars: (A and B) 1 mm; (C–K) 0.2 mm. Photo credit: Clayton C. Gonçalves.

**Figure 4:**
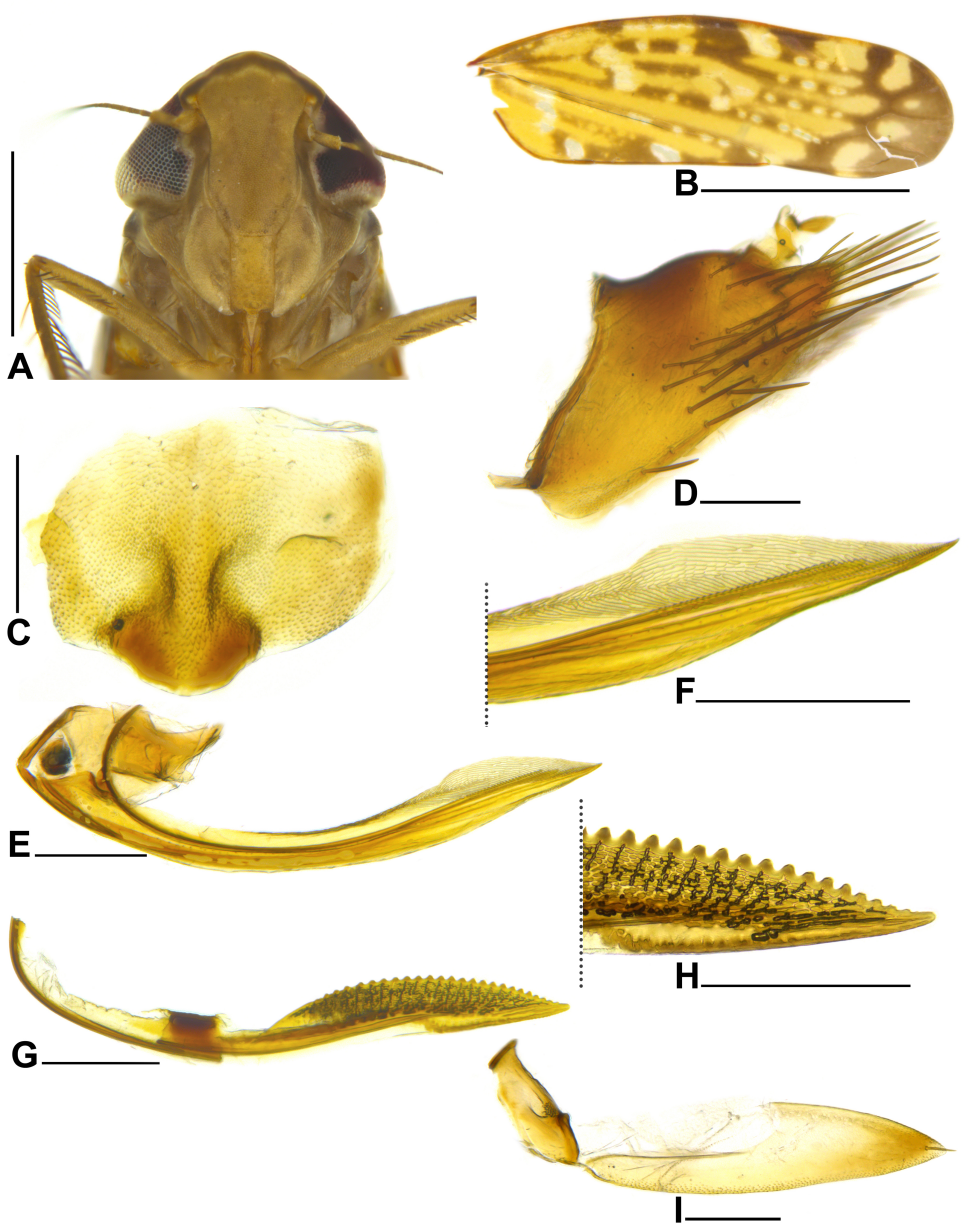
*Portanus tambopata* sp. nov., female paratype. (A) Head and anterior thorax, ventral view. (B) Forewing, dorsal view. (C) Sternite VII, ventral view. (D) Pygofer and anal tube, lateral view. (E) First valvifer and first valvula, lateral view. (F) Apical portion of first valvula, lateral view. (G) Second valvula, lateral view. (H) Apical portion of second valvula, lateral view. (I) Second valvifer and gonoplac, lateral view. Scale bars: (A and B) 1 mm; (C–I) 0.2 mm. Photo credit: Clayton C. Gonçalves.

**Type locality**. Refugio Amazonas, Madre de Dios, Peru.

**Diagnosis**. Male pygofer (Fig. 3C), in lateral view, subtriangular; posterior margin truncate, with small dorsal teeth and subquadrate ventral lobe bearing slender and acute process directed posteriorly. Aedeagus (Figs. 3H–3J) preatrium slightly sinuous; shaft enlarged at base, narrowing towards apex; apex with single bifurcated process turned ventrally, sinuous and with apices turned outwards, resembling an anchor (Fig. 3I). Male anal tube (Figs. 3C and 3K) segment X with pair of small, lateral, strongly sclerotized toothed lobes at middle third. Female sternite VII (Fig. 4C) approximately rectangular; posterior margin with prominent rounded median lobe.

**Measurements** (mm). Males (*n* = 5)/females (*n* = 2): body length, 4.3–4.8/4.6–4.7; crown length, 0.4/0.4; transocular width, 1.1/1.2; interocular width, 0.5–0.6/0.6; maximum pronotum width, 1.0–1.1/1.1; forewing length, 3.3–3.6/3.5–3.7.

**Coloration**. Crown brown; anterior margin with dark brown line; apical third with subtriangular marking between ocelli, which extends to posterior margin as a median line, pale yellow; basal two-thirds with longitudinal pale-yellow line surrounded by a reddish-brown area. Ocellus red. Face and gena pale brown and lorum ivory (Figs. 3A and 4A). Pronotum brown, with several ivory spots. Mesonotum brown; pair of lateral triangular dark-brown maculae on anterior margin; posterolateral margin ivory. Scutellum pale brown to ivory. Forewing (Figs. 3B and 4B) translucent yellowish brown; veins dark brown with alternating ivory spots; five dark brown triangular maculae along costal margin; apex dark brown. Thoracic venter ivory. Legs ivory; posterior apexes of tibia, first and second tarsomeres brown.

**Description**. Head (Figs. 3A and 4A), in dorsal view, with anterior margin angulate; crown median length approximately seven to eight-tenths of interocular width and three to four-tenths of transocular width; lateral frontal suture reaching ocellus; epicranial suture not extended to imaginary transverse line between ocelli; texture shagreen. Pronotum width subequal to head width; lateral margin angulate; posterior margin straight; texture smooth with transverse striae. Mesonotum shagreen. Forewing (Figs. 3B and 4B) with distinct venation; with three closed anteapical cells, median anteapical cell slightly longer than others. Metatibia with row AD with 9–11 long cucullate setae intercalated by 3–4 shorter setae; PD row with 10 very long cucullate setae intercalated by one smaller long cucullate seta. First tarsomere slightly longer than combined length of second and third; tarsomeres posterior margin with three, two, and zero platellae, respectively, between pair of outer slightly longer setae.

**Male genitalia.** Pygofer (Fig. 3C), in lateral view, slightly longer than high; subtriangular; posterior margin truncate, with small dorsal teeth and subquadrate ventral lobe bearing slender and acute process directed posteriorly; macrosetae distributed at median portion dorsally; microsetae at apex. Valve (Fig. 3E), in ventral view, oblong; wider than long; anterior and posterior margin convex. Subgenital plate (Figs. 3D and 3E) extending posteriorly farther than pygofer apex; apical third upturned; in ventral view, basal third with transverse unpigmented line; surface with 5–6 robust macrosetae uniseriate and many long and fine microsetae at apical half. Connective (Fig. 3F), in dorsal view, Y-shaped; anterior margin with short median basiventral triangular projection; apex truncate. Style (Figs. 3F and 3G) with apodeme (basal portion anterad of connective articulation) long, one-third of total length; apical third widened with preapical lobe elongate and robust; apex truncated with digitiform process; in lateral view, subcylindrical and sinuous. Aedeagus (Figs. 3H–3J) with long and slightly sinuous preatrium; dorsal apodeme not so sclerotized; shaft wider at base, narrowing towards apex; apex with single bifurcated process directed ventrally, with rami sinuous, half-length of shaft, with apices turned outwardly, resembling an anchor. Anal tube segment X (Figs. 3C and 3K) subcylindrical; as long as pygofer; with few denticles on ventral margin at base; with pair of small lateral, strongly sclerotized, toothed lobes at median third.

**Female genitalia**. Sternite VII (Fig. 4C), in ventral view, approximately rectangular; posterior margin with prominent rounded median lobe. Pygofer (Fig. 4D), in lateral view, higher than long; subtriangular; ventral margin twice longer than dorsal margin; dorsal margin with convex median portion; with long macrosetae concentrated at apical half; without microsetae; apex acute. First valvifer (Fig. 4E) subtrapezoidal. First valvula (Fig. 4E), in lateral view, expanded apically; ventral interlocking device located on basal third of blade; dorsal sculptured area on apical fourth, with sculpturing strigate (Fig. 4F); apex acute. Second valvifer (Fig. 4I) three times higher than long. Second valvula (Figs. 4G and 4H), in lateral view, with apical half expanded, narrowing to apex; dorsal margin with 24 separate subtriangular teeth without denticles (single specimen dissected); duct area with maculose sculpturing; ventral margin without preapical prominence or denticles; apex acute. Third valvula (Fig. 4I) with basal half distinctly narrower than apical half; microsetae distributed on ventral margin and dorsal margin near apex; one apical macroseta; apex acute.

**Remarks**. *Portanus tambopata* **sp. nov.** is very similar to *Portanus bifurcus* Carvalho & Cavichioli, 2017, both species sharing: (1) a similar color pattern; and (2) posterior margin of male pygofer truncate with ventral lobe. However, the new species can be distinguished from the latter and other *Portanus* species by its posterior margin of male pygofer lobe with subquadrate ventral lobe bearing a long and slender process directed posterodorsally (Fig. 3C) (in *P. bifurcus*, posterior margin of male pygofer lobe with ventral lobe acute without slender process) and aedeagus apex with single bifurcated process directed ventrally, with rami apices turned outwardly like an anchor (Figs. 3H–3J) (in *P. bifurcus* aedeagus apex has pair of bifurcated processes, which have apices directed ventrally).

**Etymology**. The species epithet is a reference to Tambopata National Reserve, area from where the type series was collected. The species epithet is treated as a noun in apposition.

**Material studied. Holotype**. 1 male, “**PERU**, MD [Madre de Dios], Albergue\Refugio Amazonas\12°52′30″[S]/69°24′35″[W]\231 m 28.iii.2016\D. Couceiro”, “Malaise Trap” (MUSM). **Paratypes.** 1 female, same data as holotype, except: “241 m 01.xii.2016”, “WIRED AMAZON\PROJECT\MALAISE TRAP” (MUSM); 1 female, same data as preceding, except “231 m 15.v.2016” (DZRJ-AUCH-161); 1 male, same data as holotype, except “02.x.2016” (MUSM); 2 males, same data as holotype, except “12.iv.2016; WIRED AMAZON\PROJECT\MALAISE TRAP” (DZRJ-AUCH-159-160); 1 male, same data as preceding, except “29.ii.2016\J. Grados” (Cicadell-JGA-009, MUSM).

**Checklist of Portanini from Peru**

**(1) *Metacephalus albocrux*DeLong & Martinson, 1973**

**Distribution**. Brazil (Souza, Takiya & Felix, 2017); Peru: Cusco [**New Record**], Ucayali (type locality: Pucallpa), and San Martín [**New Record**] Departments.

**Material studied. PERU**: 2 males, San Martín Prov., Concervación Mun. Zona Barreal, 23 km S Picota, in dry forest, 7°4.88′S 76°18.89′W, 335 m, Malaise, 6–15.iii.2005, M.E. Irwin and J.D. Vasquez (INHS 852,801-852,802). 2 males, Cusco, 3rd km E Quincemil, 13°13′3″S 70°43′40″W, 633 m, 20.viii-01.ix.2012, malaise, RR Cavichioli, JA Rafael, APM Santos & DM Takiya (DZRJ-AUCH-100-101). 1 male, Cusco, Puente Inambari, 13°10′53″S 70°23′06″W, 365 m 19.VIII.2012 light, APM Santos & DM Takiya (MUSM).

**(2) *Metacephalus bicornis* (Carvalho & Cavichioli, 2003)**

(Figs. 5I and 5J)

**Figure 5:**
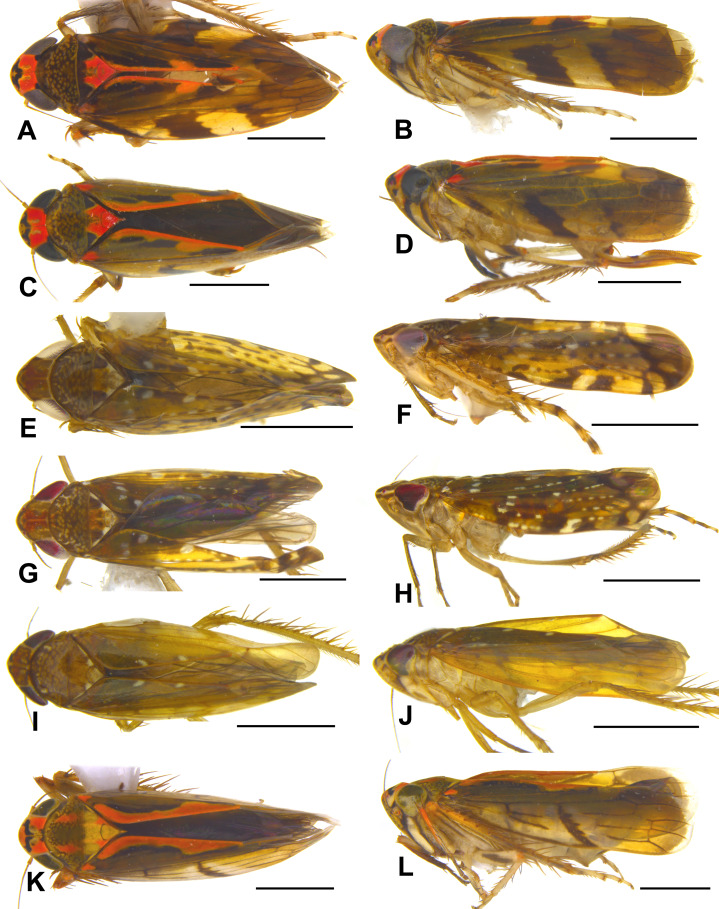
Dorsal and lateral habitus of Portanini species recorded from Tambopata National Reserve from Peru. (A and B) *Metacephalus mamaquilla* **sp. nov.**, male holotype. (C and D) *Metacephalus mamaquilla* **sp. nov.**, female paratype. (E and F) *Portanus tambopata* **sp. nov.**, male holotype. (G and H) *Portanus tambopata* **sp. nov.**, female paratype. (I and J) *Metacephalus bicornis* (Carvalho & Cavichioli, 2003), male. (K and L) *Metacephalus elegans* (Kramer, 1961), male. Scale bars: 1 mm. Photo credit: Clayton C. Gonçalves.

**Distribution**. Brazil (type locality: Vilhena, Rondônia State); Peru [**New Record**]: Madre de Dios Department.

**Material studied. PERU**: 1 male, Madre de Dios, Refugio Amazonas, Albergue, 12°52′30″S 69°24′35″W 231 m, 03.ix.2016, D. Couceiro, Malaise Trap.; Wired Amazon Project (MUSM). 1 male, same data as preceding, except 12.iv.2016 (DZRJ-AUCH-102). 1 male, same data as preceding, except 14.x.2014, PAN Trap (MUSM).

**(3) *Metacephalus eburatus* (Kramer, 1964)**

**Distribution**. Brazil (Carvalho & Cavichioli, 2009); Colombia (Freytag & Sharkey, 2002); Guyana (Felix & Mejdalani, 2016); Panama (type locality: Fort. Gulick, Canal Zone); Peru [**New Record**]: Loreto Department; Venezuela (Kramer, 1964).

**Material studied. PERU**: 2 males and 1 female, Dept. Loreto, San Juan de Pamplona, 35 km S Yurimaguas, Malaise in Oil Palm/Cacao Plantation, 6°7′38″S 76°11′26″W, 170 m, 11–18.iv.2009, malaise, G. Antón Amaya & M.E. Irwin (INHS 852,803–852,805). 1 male, same data as preceding (DZRJ-AUCH-103).

**(4) *Metacephalus elegans* (Kramer, 1961)**

(Figs. 5K and 5L)

**Distribution**. Brazil (Carvalho & Cavichioli, 2009); Colombia (Freytag & Sharkey, 2002); Peru [**New Record**]: Amazonas and Madre de Dios Departments; Venezuela (type locality: Culebra Community, Duida-Marahuaca National Park, Amazonas State).

**Material studied. PERU**: 1 male and 1 female, Madre de Dios, Refugio Amazonas, Albergue, 12°52′30″S 69°24′35″W 231 m, 03.v.2016, D. Couceiro, Malaise Trap.; Wired Amazon Project (MUSM). 1 male, Dept. Amazonas, Distr. Aguas Verdes, Bagua/Tarapoto Rd (5N) AT km 403, 5°41′23″S 77°38′13″W, 1,125 m, Malaise, 19–26.ix.2008, M.E. Irwin & G. Antón Amaya (INHS 852,806).

**(5) *Metacephalus facetus* (Kramer, 1961)**

(Figs. 6A and 6B)

**Figure 6:**
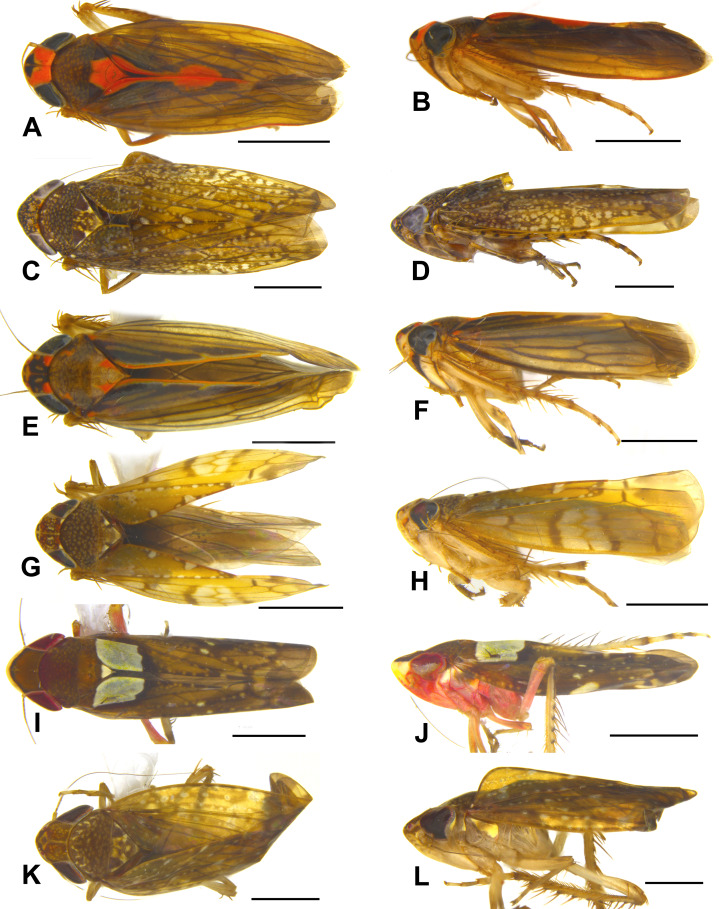
Dorsal and lateral habitus of Portanini species recorded from Tambopata National Reserve from Peru. (A and B) *Metacephalus facetus* (Kramer, 1961), male. (C and D) *Metacephalus longicornis* (Osborn, 1923), male. (E and F) *Metacephalus sakakibarai* Souza, Takiya & Felix, 2017, male. (G and H) *Metacephalus variatus* (Carvalho & Cavichioli, 2003), male. (I and J) *Portanus ocellatus* Carvalho & Cavichioli, 2003, male. (K and L) *Portanus sagittatus* Carvalho & Cavichioli, 2004, male. Scale bars: 1 mm. Photo credit: Clayton C. Gonçalves.

**Distribution**. Brazil (Carvalho & Cavichioli, 2009); Colombia (Freytag & Sharkey, 2002); Peru [**New Record**]: Amazonas, Cusco and Madre de Dios Departments; Venezuela (type locality: Upper Cunucunuma River, Tapara, Amazonas State).

**Material studied**. **PERU**: 1 male, Dept. Amazonas, Distr. Aguas Verdes, Bagua/Tarapoto Rd (5N) AT km 403, 5°41′23″S 77°38′13″W, 1,125 m, Malaise, 24-31.x.2008, M.E. Irwin & G. Antón Amaya (INHS 852,808). 1 male, same data as preceding, except 8-15.vii.2008 (INHS 852,809). 1 male, same data as preceding, except 20-27.ii.2009 (INHS 852,807). 1 male, same data as preceding, except 6-13.iii.2009 (DZRJ-AUCH-114). 1 male, Cusco, 19rd km W Quincemil, Rio Araza Tributary, 13°20′10″S 70°50′57″W, 874 m, 23-31.viii.2012, malaise, RR Cavichioli, JA Rafael, APM Santos & DM Takiya (DZRJ-AUCH-107). 1 male, Madre de Dios, Refugio Amazonas, Albergue, 12°52′30″S 69°24′35″W 231 m, 01.vi.2016, D. Couceiro, PAN Trap.; Wired Amazon Project (MUSM). 1 male, same data as preceding, except 01.xii.2016 (MUSM). 2 males, same data as preceding, except 02.x.2016 (MUSM). 3 males, same data as preceding, except 03.v.2016, malaise (DZRJ-AUCH-110-112). 1 female, same data as preceding, except 03.xi.2016, malaise (DZRJ-AUCH-113). 1 male, same data as preceding, except 09.iii.2016, 241 m, malaise (MUSM). 1 male, same data as preceding, except 12.ii.2016, J. Grados (Cicadell-JGA-001, MUSM). 3 males, same data as preceding, except 12.iv.2016, malaise (DZRJ-AUCH-104-106). 1 male, same data as preceding, except 15.xi.2016 (MUSM). 1 male, same data as preceding, except 17.x.2016 (MUSM). 1 male, same data as preceding, except 19.iii.2016, malaise, J. Grados (Cicadell-JGA-002, MUSM). 1 male, same data as preceding, except 21.xi.2016 (MUSM). 1 male, same data as preceding, except 08.iv.2018, 241m, malaise, J. Shoobridge (MUSM). 1 male, same data as preceding, except 21.vi.2017, 241 m, malaise (MUSM). 1 female, same data as preceding, except 29.iii.2017, 241 m, J. Shoobridge (MUSM). 1 female, same data as preceding, except 24.ii.2017, malaise, 241 m, J. Grados (DZRJ-AUCH-108). 1 female, same data as preceding, except 25.v.2018, 241 m, J. Shoobridge (DZRJ-AUCH-109).

**(6) *Metacephalus longicornis* (Osborn, 1923)**

(Figs. 6C and 6D)

**Distribution**. Argentina (Linnavuori, 1959); Bolivia (type locality: Sara Province, Santa Cruz de La Sierra Department); Brazil (Carvalho & Cavichioli, 2009; Felix et al., 2020); Peru [**New Record**]: Loreto, Madre de Dios and San Martín Departments; Venezuela (Kramer, 1964).

**Material studied. PERU**: 1 male, Madre de Dios, Refugio Amazonas, Albergue, 12°52′30″S 69°24′35″W 241 m, 8.iv.2018, D. Couceiro, malaise; Wired Amazon Project (MUSM). 1 male, same label, except 18.iii.2017, J. Grados (DZRJ-AUCH-119). 5 males and 2 females, Dept Loreto, San Juan de Pamplona, 35 km S Yurimaguas, Malaise in Oil Palm/Cacao Plantation, 6°7′38″S 76°11′26″W, 170 m, 11-18.iv.2009, G. Antón Amaya & M.E. Irwin (INHS 852,810-852,816). 3 males and 1 female, same data as preceding (DZRJ-AUCH-115-118). 10 males and 1 female, San Martín Prov., Concervación Mun. Zona Barreal, 23 km S Picota, in dry forest, 7°4.88′S 76°18.89′W, 335 m, Malaise, 6-15.iii.2005, M.E. Irwin and J.D. Vasquez (USNM). 2 males, same data as preceding (DZRJ-AUCH-120-121).

**(7) *Metacephalus mamaquilla* sp. nov.**

(Figs. 1, 2, 5A–5D)

**Distribution**. Peru: Madre de Dios Department.

**Material studied**. *See above*.

**(8) *Metacephalus sakakibarai*Souza, Takiya & Felix, 2017**

(Figs. 6E and 6F)

**Distribution**. Brazil (type locality: Ipixuna, Amazonas State); Peru [**New Record**]: Cusco and Madre de Dios Departments.

**Material studied**. **PERU**: 1 male, Madre de Dios, Refugio Amazonas, Albergue, 12°52′30″S 69°24′35″W 231 m, 02.x.2016, D. Couceiro, malaise; Wired Amazon Project (MUSM). 2 males, Cusco, 19rd km W quincemil, Rio Araza Tributary, 13°20′10″S 70°50′57″W, 847 m, 23-31.viii.2012, malaise, RR Cavichioli, JA Rafael, APM Santos & DM Takiya (MUSM). 2 males, same data as preceding (DZRJ-AUCH-129-130).

**(9) *Metacephalus variatus* (Carvalho & Cavichioli, 2003)**

(Figs. 6G and 6H)

**Distribution**. Brazil (type locality: Ouro Preto d’Oeste, Rondônia State); Peru: Madre de Dios (Carvalho & Cavichioli, 2009) and San Martín [**New Record**] departments.

**Material studied. PERU**: 46 males, San Martín Prov., Concervación Mun. Zona Barreal, 23 km S Picota, in tropical deciduous forest, 7°4.88′S 76°18.89′W, 335 m, Malaise, 6-15.iii.2005, M.E. Irwin and J.D. Vasquez (INHS 852,817-852,862). 10 males, same data as preceding (DZRJ-AUCH-132-141). 1 male, Madre de Dios, Refugio Amazonas, Albergue, 12°52′30″S 69°24′35″W 241 m, 18.iii.2017, J. Grados, malaise; Wired Amazon Project (Cicadell-JGA-008, MUSM). 1 male, same label, except 19.iii.2016 (DZRJ-AUCH-131).

**(10) *Portanus acerus*DeLong, 1976**

**Distribution**. Bolivia (type locality: San Esteban, Santa Cruz de La Sierra, Santa Cruz Department); Peru [**New Record**]: Loreto and San Martín departments.

**Material studied. PERU**: 1 male, Dept Loreto, San Juan de Pamplona, 35 km S Yurimaguas, Malaise in Oil Palm/Cacao Plantation, 6°7′38″S 76°11′26″W, 170 m, 11-18.iv.2009, G. Antón Amaya & M.E. Irwin (INHS 852,863). 15 males, San Martín Prov., Concervación Mun. Zona Barreal, 23 km S Picota, in dry forest, Malaise, 7°4.88′S 76°18.89′W, 335 m, 6-15.iii.2005, M.E. Irwin and J.D. Vasquez (INHS 852,864-852,878). 5 males, same data as preceding (DZRJ-AUCH-142-146).

**(11) *Portanus avis*DeLong, 1980**

**Distribution**. Peru (type locality: Sinchona [precise locality unknown]).

**(12) *Portanus bilineatus*DeLong, 1982**

**Distribution**. Peru (type locality: Sinchona [precise locality unknown]).

**(13) *Portanus boliviensis* (Baker, 1923)**

**Distribution**. Argentina (Linnavuori, 1959); Bolivia (type locality: Las Juntas, Santa Cruz de La Sierra Department); Brazil (Souza & Takiya, 2014); Peru: Vilcanota [probably Cusco Department] (Linnavuori, 1959).

**(14) *Portanus cellus*DeLong, 1980**

**Distribution**. Peru (type locality: Sinchona [precise locality unknown]).

**(15) *Portanus cephalatus*DeLong, 1980**

**Distribution**. Peru (type locality: Sinchona [precise locality unknown]).

**(16) *Portanus dentatus*DeLong, 1980**

**Distribution**. Peru: Sinchona (type locality [precise locality unknown]) and Amazonas Department [**New Record**].

**Material studied. PERU**: 1 male, Dept. Amazonas, Distr. Aguas Verdes, Bagua/Tarapoto Rd (5N) AT km 403, 5°41′23″S 77°38′13″W, 1,125 m, Malaise, 24-31.x.2008, M.E. Irwin & G. Antón Amaya (INHS 852,881). 2 males, same label data, except, 12-19.ix.2008 (INHS 852,879-852,880). 3 males and 1 female, same label data, except, 29.v-5.vi.2009 (DZRJ-AUCH-147-150).

**(17) *Portanus inflatus*DeLong & Linnavuori, 1978**

**Distribution**. Peru: Sinchona (type locality [precise locality unknown]) and Pasco Department [**New Record**].

**Material studied. PERU**: 1 male, Pasco Department, P.N. Yanachaga Chemillén, Puesto de Control Huampal, on windows, at night, 06.x.2002, 10°11′08″S 75°34′25″W, 1,050 m, R.A. Rakitov (INHS 852,882).

**(18) *Portanus ocellatu*s Carvalho & Cavichioli, 2003**

(Figs. 6I and 6J)

**Distribution**. Brazil (type locality: Sinop, Mato Grosso State); Peru [**New Record**]: Cusco and Madre de Dios Departments.

**Material studied. PERU**: 1 male, Cusco, Puente Inambari, 13°10′53″S 70°23′06″W, 365 m 19.viii.2012 light, APM Santos & DM Takiya (MUSM). 1 male, Madre de Dios, Refugio Amazonas, Albergue, 12°52′30″S 69°24′35″W 241 m, 09.iii.2016, D. Couceiro, Malaise Trap; Wired Amazon Project (MUSM). 3 males, same data as preceding, except 12.iv.2016 (MUSM). 1 male, same data as preceding, except 19.iv.2016 (MUSM). 1 male, same data as preceding, except 21.vi.2016 (MUSM). 1 female, same data as preceding, except 28.viii.2016 (MUSM). 2 males and 2 females, same data as preceding, except 02.x.2016 (DZRJ-AUCH-151-154). 1 male, same data as preceding, except 03.xi.2016 (DZRJ-AUCH-155).

**(19) *Portanus retusus*Linnavuori & DeLong, 1979**

**Distribution**. Bolivia (type locality: Lamba, Clapare (sic!) [Chapare] Province, Cochabamba Department); Peru [**New Record**]: Cusco Department.

**Material studied. PERU**: 1 male and 1 female, Cusco, Ttio, 13°31′54″S 70°53′55″W, 2,000 m, Light, 30.viii.2012, APM Santos & DM Takiya (MUSM).

**(20) *Portanus sagittatus*Carvalho & Cavichioli, 2004**

(Figs. 6K and 6L)

**Distribution**. Brazil (type locality: Ouro Preto d’Oeste, Rondônia State); Peru [**New Record**]: Cusco and Madre de Dios departments.

**Material studied. PERU**: 2 males, Madre de Dios, Mazuco, 12RD km E Mazuco, PT e Amanapu, 13°2′51.1″S 70°20′45.9″W, 382 m, malaise, 18-22.viii.2012, R Cavichioli, JA Rafael, APM Santos & DM Takiya (MUSM). 2 males, dame data as preceding (DZRJ-AUCH-156-157). 1 male, Cusco, 3rd km E Quincemil, 13°13′3″S 70°43′40″W, 633 m, 20.viii-01.ix.2012, malaise, RR Cavichioli, JA Rafael, APM Santos & DM Takiya (MUSM). 1 male, Madre de Dios, Refugio Amazonas, Albergue, 12°52′30″S 69°24′35″W 231 m, 03.v.2016, D. Couceiro, Malaise Trap.; Wired Amazon Project (MUSM). 1 male, same data as preceding, except 241 m, 21.vi.2017 (DZRJ-AUCH-158).

**(21) *Portanus tambopata* sp. nov.**

(Figs. 3, 4, 5E–5H)

**Distribution.** Peru: Madre de Dios Department.

**Material studied**. *See above*.

**(22) *Portanus uhleri*Kramer, 1964**

**Distribution**. Argentina (type locality: Loreto, Misiones Province); Peru [**New Record**]: San Martín Department.

**Material studied. PERU**: 17 males and 1 female, San Martín Prov., Concervación Mun. Zona Barreal, 23 km S Picota, in dry forest, 7°4.88′S 76°18.89′W, 335m, Malaise, 6-15.iii.2005, M.E. Irwin and J.D. Vasquez (INHS 852,883-852,900). 5 males, same data as preceding (AUCH-162-166).

## Discussion

The present revision of leafhopper material collected in Tambopata National Reserve, as well as, Peruvian material from different collections, resulted in the finding of two undescribed species and a great number of new species records from Peru. Portanini, with nine species recorded until the present work, now have 22 species recorded for this country. The majority of Portanini species are only known from original male genitalia drawings and/or descriptions. For this reason, pictures of dorsal and lateral habitus of species of Portanini collected from Tambopata National Reserve are provided to help in the identification of specimens for future studies with this tribe.

Cicadomorpha is an understudied group in South America, with representatives of several lineages not having been studied for decades or centuries, and those that are currently being studied are far too diverse and have a great number of undescribed species (Freytag & Sharkey, 2002; Costa & Lozada, 2010; Bartlett et al., 2018). For the particular case of leafhoppers of Peru, only two checklists exist, recording 634 species of some subfamilies of Cicadellidae (Lozada, 1992, 1997), however, this number seems to be underestimated due to the lack of complete studies for this group that could reveal a much higher diversity (Costa & Lozada, 2010). The same probably applies to the currently 679 leafhopper species recorded from Colombia (Freytag & Sharkey, 2002). Given the size of the country, even the approximately 1,800 leafhopper species recorded from Brazil is also considered to be highly underestimated (Takiya et al., 2020).

## Conclusions

This study adds to the knowledge of leafhoppers from the Neotropical region. It more than doubles the number of portanine leafhoppers recorded from Peru with the description of new species, new records, and habitus photos of Portanini specimens. Our results indicate the necessity of more taxonomic studies to better document the biodiversity from this megadiverse leafhopper region.

## Supplemental Information

10.7717/peerj.10222/supp-1 Supplemental Information 1 Measurements (in mm) of *M. mamaquilla* sp. nov. and *P. tambopata* sp. nov. Raw data of total body length, forewing length, interocular width, transocular width, maximum pronotum width, and crown length for each specimen in type series. Asterisk (*) denotes holotype.

## Additional Information and Declarations

### Field Study Permissions

The following information was supplied relating to field study approvals (i.e., approving body and any reference numbers): Specimen collection at Tambopata Reserve were made under the permit N° 301-2015 and N° 019-2017 SERFOR/DGGSPFFS given by the Servicio Forestal y de Fauna Silvestre—SERFOR-Perú.

### Data Availability

The following information was supplied regarding data availability: Raw measurements of type specimens are available as a Supplemental File. A list of type material for each described species is given in the article and those are deposited in: Museo de Historia Natural, Universidad Nacional Mayor de San Marcos, Lima (MUSM) and Coleção Entomológica Prof. José Alfredo Pinheiro Dutra, Instituto de Biologia, Universidade Federal do Rio de Janeiro, Rio de Janeiro (DZRJ). Other (non-type) material studied belong these two institutions and Insect Collection, Illinois Natural History Survey, Champaign (INHS). As MUSM does not have a specimen database, those specimens studied deposited there, which were collected by Juan Grados were given the following collector numbers Cicadell-JGA-001-MUSM–Cicadell-JGA-009-MUSM. Others should be located by their label data information given in the article. Specimens studied deposited at DZRJ are databased as DZRJ-AUCH-100–DZRJ-AUCH-166 and those at INHS as INHS 852,801–INHS 852,900. Peruvian specimens were made available by C. Dietrich (INHS) and Tambopata specimens were made available by J. Grados (MUSM), team leader of the project “Discovery new species” (Wired Amazon project).

### New Species Registration

The following information was supplied regarding the registration of a newly described species: Publication LSID: urn:lsid:zoobank.org:pub:EEA39E0C-D2C0-494C-B1D7-F7E6B3D818CD *Metacephalus mamaquilla* sp. nov. LSID: urn:lsid:zoobank.org:act:8CD03270-1760-4962-8D9E-26F639FB8E04 *Portanus tambopata* sp. nov. LSID: urn:lsid:zoobank.org:act:9C799CBA-FD0C-4DB3-931D-7FB7ECA440E6.
